# Combining Resource, Structure and Institutional Environment: A Configurational Approach to the Mode Selection of the Integrated Healthcare in County

**DOI:** 10.3390/ijerph16162975

**Published:** 2019-08-19

**Authors:** Li Zhu, Zixuan Peng, Lihang Liu, Shuang Ling

**Affiliations:** 1 School of Public Administration, Central South University, Changsha 410083, China; 2 School of Public Health, University of Toronto, Toronto, ON M2J 4A6, Canada

**Keywords:** Integrated Healthcare, Integrated Healthcare in County (IHC), resource heterogeneity, governance structure, institutional normalization

## Abstract

Integrated healthcare has received considerable attention and has developed into the highly important health policy known as Integrated Healthcare in County (IHC) against the background of the Grading Diagnosis and Treatment System (GDTS) in rural China. However, the causal conditions under which different integrated health-care modes might be selected are poorly understood, particularly in the context of China’s authoritarian regime. This study aims to identify these causal conditions, and how they shape the mode selection mechanism for Integrated Healthcare in County (IHC). A theoretical framework consisting of resource heterogeneity, governance structure, and institutional normalization was proposed, and a sample of fifteen IHCs was selected, with data for each IHC being collected from news reports, work reports, government documents and field research for Fuzzy-sets Qualitative Comparative Analysis (fsQCA). This study firstly pointed out that strong governmental control and centralization are necessary conditions for the administration-oriented organization mode (MOA). Additionally, this research found three critical configured paths in the selection of organizational modes. Specifically, we found that the combination of low resource heterogeneity, weak governmental control, centralization, and normalization was sufficient to explain the selection path of the insurance-driven organization mode (MOI); the combination of low resource heterogeneity, strong governmental control, centralization, and normalization was sufficient for selecting MOA; and the combination of weak governmental control, weak centralization, and weak normalization was sufficient for selecting the contractual organization mode (MOC). Our study highlighted the necessity and feasibility of constructing different IHC modes separately and promoting their development gradually, as a result of the complex relationships among the causal conditions described above, thus helping to optimize the distribution of health resources and integrate the healthcare system.

## 1. Introduction

At the end of the 20th century, Shortell (1993) [1] proposed the concept of integrated healthcare, advocating the provision of proper one-stop health services for patients in accordance with their health conditions and financial status, in order to reduce the cost and improve the quality of health services. This concept has received considerable attention in the field of global health, and has been put into practice in rural areas with a variety of organizational forms, such as Integrated Rural Health Networks and Vertically Integrated Health Systems [2,3]. Subsequently, this has been developed into the highly significant health policy known as Integrated Healthcare in County (IHC), against the backdrop of the Grading Diagnosis and Treatment System (GDTS) in rural China. Generally speaking, Integrated Rural Health Networks are more effective and more widely applicable for connecting their members than either systems or alliances, since “systems” are formal groups that imply a common ownership of almost all of their members, while “alliances” indicate loose relationships among independent health service providers [4]. In practice, Vertically Integrated Rural Health Networks (VIRHN), which are a vertical implementation of the Integrated Rural Health Network concept, have been employed widely in the United States, in the form of clinical integration, functional integration, physician integration, and financial integration, and have become a strategic choice in the rural US for coping with increasingly fierce market competition [4,5,6]. In addition, IHC is a policy-orientated organizational form led by local governments and has become a critical organizational form for restructuring the GDTS and reforming the healthcare service supply system in rural China. However, consensus has not yet been reached with regard to the selection of specific organizational forms, and both VIRHN and IHC are dependent on the administrative [7], demonstrating the coexistence of both “structural similarities” and “inconsistencies”. In the context of China’s authoritarian regime, an implementation of IHC characterized by “vertical cooperation, horizontal complementarity and competition” has flourished across most counties, with widely implemented organizational modes differing in their level of integration due to local conditions. Therefore, studies identifying the relationship between mode selection and the corresponding conditions under which the selections are made are needed.

There is plenty of evidence to suggest that both vertical integration and organizational networks are dynamic processes that are impacted by various short-term and long-term institutional factors [8,9]. More specifically, resource conditions, leaders’ willingness, and environmental relations within the organizations can influence the forms of governance selected in the construction of Integrated Rural Health Networks [2,5,10,11]. However, no single factor is able to explain the formation mechanism. Practical implementations of the IHC policy are still typically in their pilot stages, and most practitioners have faith in a priori hypothesis that resource heterogeneity promotes organizational integration, and that more integrated forms will perform better than more loosely organized ones; thus, there is always the intent to construct more integrated organizational forms. In this sense, this study should first analyze the fundamental resource conditions specific to the implementation of IHC. Additionally, in the context of China’s authoritarian regime, the guiding role played by health authorities, and even the government as a whole, should be considered [12]. In addition, practically speaking, county-level hospitals lead the coordination of all the health service providers, and the internal relationships among them also need to be considered [13]. Moreover, the institutional environment undoubtedly provides paths for organizational behavior, thus influencing the evolution process of organizational mode through continuous interaction [14], meaning that the effectiveness of the issued policies also reflects institutional normalization in certain counties, and this should also be taken into account [15]. In other words, IHC as a policy process is primarily affected by the interactions among health service providers within the IHC, and is also impacted by relationships with external actors, most significantly with the local government, and even by the logic by which the institutional environment is selected and defined. Thus, a systematic theoretical framework concerning causal conditions will be proposed, although the influence of resource conditions, governance structures and institutional environments, as well as the actual mechanism of IHC, remains to be confirmed by means of empirical testing. 

In this article, we first review the literature and propose a systematic analytical framework from the perspective of resource dependency theory. Then, Fuzzy-Sets Qualitative Comparative Analysis (fsQCA), which is a comparative configurational method and is appropriate for carrying out complex causality research with medium-sized samples, was performed in county areas that are implementing the IHC policy and reforming their health grading systems against the backdrop of an authoritarian environment. Data were collected from 2017 to 2019 in order to verify the influence of resource conditions, governance structure, and the institutional environment, as well as to reveal the mechanism for the selection of IHC mode selection. Finally, our findings and prospects are presented for reference. 

## 2. Literature Review and Hypothesis Development

### 2.1. Definition and Modes of IHC

In the context of “Healthy China”, the policy process known as IHC has gradually gained recognition throughout whole society because of its concepts of “disease prevention and health promotion”. The State Council Office has successively issued the “Guiding Opinions on Promoting the Construction of Grading Diagnosis and Treatment System”, the “Guiding Opinions on Promoting the Construction and Development of Integrated Healthcare”, and other policy documents. In the context of such a policy environment, IHC led by local governments has become the organizational form by which it has been possible to explore the restructuring of the GDTS, the defragmentation of the health service supply system, and the optimal allocation of medical and health resources. Some scholars have defined IHC as an organizational form that integrates health resources while implementing united management in order to achieve the policy goals of the GDTS within a county [16,17]. Other scholars have argued that IHC is an operational form of Integrated Healthcare and Medical Associations (MA) at the county level [18]. As mentioned above, our study argues that IHC is a shared organizational form founded on the basis of general recognition and tolerance of differences among three or more health service providers within a county, in the pursuit of coordination of interests and sharing of responsibilities. Generally, the county-level hospital occupies the most resource conditions, and it is inevitably included in any given IHC.

According to the institutional implementation of GDTS and the policy process of Integrated Healthcare in China, the property rights of the health service providers in an IHC are maintained as before, and all members have qualifications as independent corporations for implementing IHC. Thus, we first excluded property-based associations. Additionally, as Fang D. said in “Building Virtual Government: Information Technology and Institutional Innovation”, modern information technology cannot reorganize internal structures, because reorganization can only be achieved by combining modern information technology with organizational structures and institutional arrangements. Therefore, the virtual integration form, which is an emerging form of integration, was also excluded from the rural healthcare systems. As Proven pointed out, network structures can mainly be categorized as Lead Organization Networks, Network Administrative Organizations, or Participant-Governed Networks [19], with the classification being based on the relations among the member organizations. In this study, we denoted the IHC organization modes as the Insurance-driven Organization Mode (MOI), the Administration-oriented Organization Mode (MOA) and the Contractual Organization Mode (MOC) with reference to the manner in which GDTS and IHC were implemented, as well as the classification criteria of the network structure (Table 1).

In our research, the classification principle was based on whether vertical integration involves the collective allocation of human, financial and material resources, and the manner in which the resources are distributed. As shown in Table 1, the Insurance-driven Organization Mode (MOI) involves the allocation of medical insurance, which is difficult to carry out. The Administration-oriented Organization Mode (MOA) involves a united but limited management of the human, financial and material resources for health service providers. In addition, the Contractual Organization Mode (MOC), which is relatively pervasive, doesn’t involve the allocation of such resources. All three modes are widely implemented in rural China, and this classification conforms to the policy implementation of IHC.

### 2.2. Hypothesis Development

Current studies focus on the fundamental conditions, such as the resource conditions, that determine mode selection. Some empirical research has demonstrated that the greater the resource heterogeneity among member organizations, the more likely that member organizations will be inclined to choose the contractual organization, and vice versa [20]. However, little attention has been paid to the impact of resource heterogeneity on IHC mode selection. Thus, this study will first identify the relationship between resource heterogeneity and mode selection in the context of health system bureaucracy.

#### 2.2.1. Resource Heterogeneity

Resource dependency theory emphasizes organizational power, regarding organizations as political actors and believing that organizations’ strategies are related to the organizational behaviors of resource acquisition and influencing other organizations. According to resource dependency theory, health service providers are greatly affected by their human, material, financial and technical resources; thus, they vertically integrate in order to obtain heterogeneous knowledge, technology, and other resources [21,22]. As Kanter (1988) [23] and Knott (2003) [24] stated, organizations with heterogeneous resources can innovate through resource reorganization. In our study, this kind of organizational innovation is examined in reference to the policy implementation of IHC. Based on a systematic investigation of IHC, this study defines resource heterogeneity as distinctive and diversified resource endowments among three or more organizations, and the heterogeneity is formed by the interaction among health service providers that represent a certain number of practitioners, sick beds, and medical devices.

In accordance to the institutional arrangement of GDTS, county-level hospitals are mainly responsible for disease treatment, whereas health service providers at the township-level (including village clinics) are mainly responsible for disease prevention and health promotion. Obviously, there are differences between county-level hospitals and township-level health providers; thereby, they are more likely to integrate into a cooperative organizational form. However, specific forms need to be studied further, because each county’s social economic level varies substantially, and the practitioners, sick beds and medical devices owned by each health service provider vary significantly in each IHC. In this study, we will interpret the possible effects of resource heterogeneity in two dimensions and with respect to three indicators by referring to an empirical study in Taiwan [25] (Table 2).

#### 2.2.2. Governance Structure

According to resource dependency theory, the interdependent relationship among multiple actors leads to external control over other organizations, thus forming a specific power structure. In the view of the Actor-Centered Institutionalism [26,27], county-level health authorities and other relevant governmental departments (including the financial department, the human resources and social security department, etc.), health service providers, village clinics, village committees and the like interact in the policy practice of IHC. In addition, the power structure formed by the interaction of those multiple actors will affect the external relationships, as well as the specific design of the internal relationships through the process of the health system reform [28]. Evidently, the role of government, and the degree of centralization and formalization, will affect the organizational formation and its effectiveness [29,30,31], and there must be certain compatible relationships between the above factors and the modes of governance [32]. More specifically, with respect to the external dimension, the construction of a relatively integrated network makes claims of external legitimacy, and may have been mandated by the organizational and fiscal support agents [19]; while with respect to the internal dimension, agencies embedded in a centralized network are better able to coordinate network members, leading to greater integration [32]. Admittedly, neither the external structure nor the internal structure can be immediately transformed through the implementation of IHC, and this lag will inevitably affect the actors’ resource conditions, as well as the resource heterogeneity among them. In this sense, it is assumed that there exist interactive effects between resource heterogeneity and governance structure, and that resource heterogeneity needs to be placed under specific governance structures in order to explore the actual impact of resource heterogeneity on IHC mode selection.

**Figure 1:**
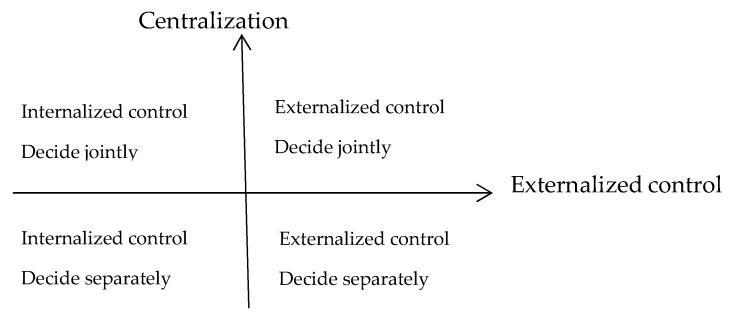
Analytical dimensions of governance structure.

In recent years, explosive research has been conducted concerning the relationship between integrated healthcare organizations and governance structures [33], and our study examines the mechanisms by which resource heterogeneity, governance structure, and their configurations affect mode selection in IHC. As mentioned above, we subscribe to the view that governance structure not only refers to the power structure between government and IHC, but also refers to the dynamic relationships among the members. Thus, governance structure will be examined from a two-dimensional perspective with respect to both the externalized control exerted by the local government and the centralization exerted among health service providers within an IHC (Figure 1).

#### 2.2.3. Normative Effectiveness

As mentioned above, a given IHC mode is the social result of the interaction between health service providers in a given institutional environment. From a perspective of institutionalism, the institutional environment provides the logic by which organizational behavior operates and influences the formation of the organization mode through continuous interaction with other organizations [34,35,36]; complex organizations such as hospitals that rely heavily on institutional and technical conditions inevitably respond to their institutional environment [37]. In the context of “Healthy China” and IHC policy, the institutional elements, which include the regulative, normative and cultural-cognitive elements in the IHC policy documents, promote the formation of the organizational field, and constantly balance the relationship between governmental authority and the independence of health service providers. In general, the implementation of a tightly integrated IHC will be carried out in reference to the ownership of the health service providers, indicating the need for a more standardized institutional environment, and vice versa. Meanwhile, the institutional resources, which mainly consist of policy resources and organizational resources in a given institutional environment, have already been absorbed by health service providers and are present in the form of either service resources or technical resources. Furthermore, the external environment directs the implementation of IHC and the corresponding governance structure, to some extent [27].

In a word, the IHC is affected by the institutional environment in the form of the fundamental effectiveness of resource conditions and the structural efficacy of the governance structure. Hence, a systematic analytical framework is proposed to explore the actual mechanism of mode selection.

#### 2.2.4. Theoretical Hypothesis

In summary, IHC has undergone systematic change influenced by the institutional environment of GDTS, and presents different forms under heterogeneous resource conditions and different governance structures. An analytical framework consisting of resource conditions, governance structure and the institutional environment based on resource dependency theory is proposed. This study hypothesizes that the resource heterogeneity, a fundamental condition, together with the appropriate governance structure and the specific institutional environment, influences the mode selection of IHC. Thus, the research proposes the following:

**Hypothesis** **1.** *The greater the resource heterogeneity among member organizations, the more likely the member organizations are to be inclined to choose a relatively more integrative organizational mode, and vice versa.*

**Hypothesis** **2.** *A relatively more integrative organizational mode is formed in accordance with stronger governmental control and higher degree of centralization.*

**Hypothesis** **3.** *A more tightly integrated organizational mode is incompatible with a more standardized institutional environment, and vice versa.*

**Hypothesis** **4.** *A relatively more integrative organizational mode is incompatible with high resource heterogeneity, strong governmental control, centralization, and highly normalized institutional environment, and vice versa.*

In this study, we propose that a single causal condition is insufficient to explain the mechanism of mode selection, and certain specific relationships are left for subsequent verification.

## 3. Materials and Methods

### 3.1. Study Method

Since the policy practice of IHC is still at the pilot stage, and the influencing factors are relatively complex, a quantitative analysis exploring the mode selection mechanism and verifying the relationship among the mentioned causal conditions (resource heterogeneity, governance structure, institutional normalization, and their configurations) on a certain number of samples is urgently required. Quantitative analysis methods are suitable for analyzing simple linear relationships within a large sample size, while qualitative analysis methods are appropriate for analyzing complicated phenomena with a small number of cases. However, the number of available samples is limited, which limits the analytical effectiveness of traditional quantitative research methods; and the time-consuming and laborious investigation required for qualitative methods might reduce the feasibility of the research when there is a medium sample size. Therefore, neither quantitative nor qualitative analysis were appropriate for tackling the relatively complex research problem in our study. To address the dilemma mentioned above, our study proposes employing Qualitative Comparative Analysis, which is a configurational comparative method that is appropriate for carrying out complex causality research with medium-sized samples [38].

### 3.2. Research Setting

According to necessity and availability, fifteen IHCs were selected in ten counties from across the eastern, middle and western regions (the names of the places were left anonymous). The preliminary database was formed after browsing related information on the Internet. Afterwards, we first investigated IHCs in Changsha city in Hunan province, and then we surveyed IHCs in other counties based on recommendation. The ultimate database (see Table 3) of this research includes 6 MOCs, 7 MOAs and 2 MOIs in different county regions, and the statistical analysis of the sample cases is presented in Appendix A.

Specifically, XSL and XXW explored health system reform earlier than the other counties; XWC (there are different methods of implementing IHC in different counties under the framework of the GDTS and IHC policies. For instance, IHC in XSS explores reform throughout county’s entire health system, with the County People’s Hospital acting as the leader; IHC in XWC explores health system reform with the County People’s Hospital, County Traditional Chinese Medicine Hospital and County Maternal and Child Care Service Centre jointly constructing a health service network characterized by vertical cooperation and horizontal competition. In our study, we selected two IHCs for analysis in XWC county) and XLJ are typical eastern counties where county-level hospitals support the development of primary healthcare providers; XNX, XCS and XTJ are typical middle counties that set medical business as the link and promote cooperation among health service providers in the IHC through contracts or informal agreements; XPJ and XSS follow an organizational mode in which the “medical insurance fund” acts as the interest link. These three kinds of organizational modes also demonstrate obvious differences, and are representative enough for our research. In general, these three modes are the social products of the health system bureaucracy supporting the policy implementation of GDTS, and they differ slightly in their macro institutional environment. At present, the support for health service providers varies in different administrative regions, and there are significant differences in resource conditions among IHC members in terms of human, financial, material and management resources, which ensures the differentiation of our cases. The discrepancies mentioned above help to analyze and compare the causal conditions and ultimately to obtain objective research results.

### 3.3. Data Collection

From 2017 to 2019, members of the research team collected data related to the sample cases and conducted follow-up investigations. Data collection was conducted in two main ways: Firstly, 10 of the IHCs were studied on the basis of semi-structured interviews and observations, due to limited accessibility. Field research supported by “the Graduate Survey Project of Central South University” and “the Fundamental Research Funds for the Central Universities of Central South University” was carried out from 2018 to 2019. Because of the transparent government structure in China, we received the opportunity to interview officials in the health authorities, the heads of the health service providers, and other social actors participating in the implementation of IHC in 10 of the IHCs. Interviewees were selected based on the principles of necessity and availability. Since the investigations are mainly with reference to the inter-organizational issues within counties, which are complicated, a relatively detailed outline concerning the external control exercised by the government in the implementation of IHCs, internal centralization, division of responsibility, and the implementation process of institutions was produced for the purpose of the semi-structured interviews.

Secondly, all of the IHCs were also evaluated on the basis of policy documents, working papers, and information lawfully disclosed by government between 2017 and 2019. When investigating the subjective influencing factors in the implementation of IHC, we used the triangulation method to verify the collected data. In terms of the timestamp, the numbers determined for practitioners, sick beds, and medical devices was true at the time just before the evaluation of the IHCs was conducted, and the collection of indicators for external control, centralization, and the normative effectiveness of policy documents in implementing IHC took place when the evaluation of each IHC had already been conducted.

Subsequently, data collected pertaining to each IHC from news reports, work reports, government documents and field research were viewed and sorted according to topics from the interview outlines in our database. All of the materials and the related data contribute to the systematic analysis of the influence of the resource condition, governance structure and institutional environment and the mode selection mechanism of IHC.

### 3.4. Measurement of Variables

#### 3.4.1. Outcome Variables

As shown in Table 1, MOI is a cooperative organization mode with the “medical insurance fund” acting as the link. In this cooperation mode, all health service providers are allocated a proportion of the medical insurance fund, thus forming a community with “interests” acting as the link. The MOA is another kind of cooperative organization mode, in which councils (or other agencies) conduct united management of health service providers. In general, hospitals at the county level implement a unified but limited management of the human, financial and material resources of health service providers at the township level by merging, hosting and branching. The MOC organization mode establishes cooperative relationships on the basis of contracts or informal agreements, and is also a relatively pervasive organization mode. In order to explore the causal conditions and their configurations in the IHC mode selection process, a binary measurement method is used for the outcome variables (see Table 4).

#### 3.4.2. Conditional Variables

Conditional variables include resource heterogeneity, governance structure and normative effectiveness. Specifically, resource heterogeneity is defined by the heterogeneity of service resources and technical resources. Governance structure is analyzed based on the balance between governmental control and internal control, as well as the balance between centralization and decentralization. Normative effectiveness is used to characterize the degree of normality of policy documents. The specific definition criteria are as follows:

Resource Heterogeneity

As shown in Table 4, this study depicts resource heterogeneity based on the number of practitioners (including assistant practitioners), open sick beds, and available medical equipment. In the first place, we measure the heterogeneity of technical resources based on the configuration of medical equipment in an IHC, and this equipment will include ambulances, biochemical instruments, electrocardiograms, B-ultrasounds, color ultrasounds, X-ray machines, DR/CR, CT and MRI, etc. With reference to the previous research carried out by Teachman (1980) [39] and Meyer and Goes (1988) [40], the specific calculation formula is as follows:

entropy_techi=[∑Pij×Ln(1/Pij)]/LnI

Specifically, *j* represents medical devices owned by the *ith* hospital, *Pij* indicates the proportion of medical devices located in the *ith* hospital; *LnI* indicates the theoretical maximum value of entropy. According to the definition of entropy, which indicates the degree of internal chaos in a system, the more chaotic a system is, the more even its distribution. In other words, the smaller the value of entropy*_techi*, the greater the heterogeneity of technical resources.

In addition, this study measures heterogeneity of service resources based on the number of licensed physicians (including assistant practitioners) and open sick beds in the calculation formula described above. Then, Principal Component Analysis (PCA) is used to determine the variable of resource heterogeneity. This comprehensive indicator of resource heterogeneity is determined in order to achieve the required balance between the number of cases and the number of conditions [38].

Subsequently, we calibrated the calculated value, which is a continuous value from 0 to 1, and set the full membership, the crossover point and the full non-membership threshold as 0.83, 0.73 and 0.57, respectively, after many attempts.

Governance Structure

In this research, governance structure is defined in terms of the dimensions of governmental control and centralization. On the one hand, governmental control is primarily used to describe the manner in which local government affects the IHC at an organizational and fiscal level, and the extent of this intervention. In the context of a bureaucratic health system, health system reform is promoted by the health department and exhibits governmental control to some extent. In general, voluntary cooperative networks that are established among health service providers in a county are able to receive little if anything in the way of governmental support, and the governmental control over them is quite low. Actually, health system reform involves cooperation between the health department and related departments, such as the finance department and the human resources and social security department, and the administrative power of the health department is relatively limited. The administrative power of working groups led by main government officials (often referred to as the permanent member of a committee in China) is significantly stronger than that of the single health department. In addition, working groups led by the heads of county-level government have the strongest administrative power and are more likely to receive organizational and financial support. Therefore, a quartering method (“1/0.67/0.33/0”) is employed to portray the degree of governmental control.

On the other hand, centralization is inferred on the basis of the authority exercised by the leading hospital over the member organizations in terms of the allocation of human, financial and other resources. According to the “Guiding Opinions on Promoting the Construction of Grading Diagnosis and Treatment System”, the “Guiding Opinions on Promoting the Construction and Development of Integrated Healthcare” and related implementation plans that have been publicly released, most health service providers in IHCs have maintained their previous functions and responsibilities, and explored establishing cooperative relationships with medical businesses, management or interests. In fact, the power of management of human, financial and material resources over health service providers is limited. For instance, although the health authorities at the county level having explored the trasnferral of power over personnel to county-level hospitals (i.e., recommendation and recruitment power), some of them have retained the power to inspect and appoint the heads of the health service providers. In this case, the appointment power of the leading hospital over the health service providers is limited. Therefore, this research focuses on the analysis of the limited power to allocate and distribute human, financial and material resources among health service providers. Undoubtedly, the authority of the leading hospital over the member organizations varies by region and time. As shown in Table 5, we also use the quartering method (“1/0.67/0.33/0”) to visualize the value of centralization within a specific time frame (Table 5).

In terms of value, we see that governmental control of the working group led by the head of a county-level government presents greater effectiveness than the other two working groups, and governmental control in a voluntary cooperative network is the least effective. Therefore, governmental control of the working group led by the head of county-level government is scored as “1”, the control in a voluntary cooperative network is scored as “0”, and control such as in the other two working groups are scored as “0.67” and “0.33”, respectively. The scoring of Centralization is identical to that of Governmental Control.

Normative Effectiveness

According to institutional theory, regularity, normality and cultural cognition constitute the three basic elements of normative effectiveness, which affects to a large extent the selection of organizational mode; furthermore, the normative effectiveness of policy documents can be analyzed based on policy texts. In accordance with the “Notice of the General Office of the Chinese State Council on Strengthening the Formulation, Supervision and Management of Administrative Normative Documents” (State Council 2018, No. 37), internal institutions of governmental departments are not allowed to issue normative documents, and administrative normative documents that have not been publicly released shall not be used as the basis on which to enforce administrative law. To this end, this article analyzes the normative effectiveness via a text analysis of the health policy documents issued by county-level governments, and we ensured that the selected documents had been publicly issued. (Although policy texts in XCS/XNX/XLY/XTJ are issued by the health departments, these are publicly issued policy documents. In addition, because of the lack of policy texts issued by the county-level government, the analysis of these documents is regarded as acceptable.)

The existing literature studying the effectiveness of policy texts generally analyzes the normative effectiveness of policy documents by calculating the occurrence of specified restrictive words, such as prohibition (forbidden), necessity, no, should, norms, regulations, standards, etc. [41]. In the coding phase, we adopted the above-mentioned method and tried to ascertain the membership of the selected samples on the basis of the proportion of regulatory elements, normative elements and cultural-cognitive elements in the institutional elements. Based on this textual analysis, we found that most implementation plans are led by regulatory and normative elements, and that the occurrence of these elements varies from county to county. Therefore, this study defines the degree of normativity of sample cases with reference to the occurrence of regulatory and normative elements in the policy text, setting thresholds for normative effectiveness based on many attempts and with reference to an extensive database of our related research. The normative effectiveness of the sample case is calibrated by the occurrence of specified restrictive words. Specifically, when the occurrence is no less than 22, it will be calibrated as “1”, signaling full membership, and when the occurrence is below 8, it will be calibrated as “0”, denoting full non-membership. Furthermore, the crossover point is set as 15 in order to ascertain the sample cases’ normative effectiveness.

In conclusion, in accordance with the analytical requirements of fsQCA, the selected 15 IHC samples are measured (see Table 4 and Table 5), and the resulting data matrix (in light of the definition of entropy, the greater the RH value, the lower the degree of resource heterogeneity; by this logic, the greater the calibrated RH value, the lower the degree of resource heterogeneity) is shown in Appendix B.

## 4. Results

### 4.1. Analysis of the Necessity of the Single Causal Condition

Firstly, an analysis of the necessity of a single causal condition for each organizational mode was conducted, and it was necessary to weigh their explanatory power by measuring the ratio of consistency and coverage (Table 6). In our research, the GSC and GSD of MOA both possess a consistency ratio of 1.00, and they carry coverage ratios of 0.73 and 0.78, respectively, which means that these two conditions are necessary to MOA. In contrast, other causal conditions are insufficient to explain the mode selection of any organizational mode, because their consistency ratios are less than 0.75. For this reason, it is necessary to carry out analysis of the combinations of causal conditions.

### 4.2. Analysis of Combinations

Based on the analytical results of the configurations of causal conditions, the organization mode selection paths of IHCs are complex and diversified, and the selection paths vary from one organization mode to another. By analyzing all the paths whose consistency scores were no less than 0.75, it was found that the MOI, MOA and MOC possess 3, 9 and 10 paths, respectively. More specifically, the paths of MOI point to the combination of “~GSC and INS”, as well as to their relations with other causal conditions. The paths of MOA are more complicated, showing combinations with multiple characteristics. In addition, of the paths for MOC exhibit the characteristic of multiple combinations of the negated causal conditions. In this section, we only list the combinations of causal conditions when their consistency is no less than 0.75; these are shown in Table 7 and Table 8. The analysis of the combinations does not include the analytical results of the single causal condition.

### 4.3. Truth Table Analysis

In order to obtain a relatively simple but sufficient explanation, a truth table ws constructed using fsQCA software, and a statistical analysis was performed using the Quine-McCluskey Algorithm. During the calculation process, we deleted any necessary and sufficient conditions. We set the value as 1 when the sample frequency was no less than 1 and the consistency was no less than 0.80; otherwise the value was set as 0. Then the “Standard Analysis” and “Specify Analysis” programs were used to obtain the complex solutions, the parsimonious solutions, and the intermediate solutions. On the basis of the fsQCA, the intermediate solution was based on the theoretical and practical knowledge of the researchers, incorporating meaningful logical remainders (logical remainders refer to combinations of causal conditions that were not observed in practical cases, but which exist logically), and this always performed better in practice than the complex solution and the parsimonious solution [38]. For this reason, this study will use the intermediate solution as an important basis for interpretation, and the intermediate solution for the different organization modes can be seen in Table 9. As shown in the truth table, the intermediate solution and the complex solution (i.e., the solution without the logical remainders) exhibit the same result, and the coverage of these two solutions remains around 0.50. For this reason, it is necessary to adjust the consistency value and re-code our truth table. By setting the consistency value to 0.75 (when defining the consistency threshold of MOC, a consistency score should be assigned that is no less than 0.74 to 1; when defining the consistency threshold of MOA, a consistency score should be assigned that is no less than 0.70 to 1; this is in accordance with the characteristics of the sample cases in our research), the complex solution, parsimonious solution, intermediate solution and the consistency and coverage of different organization modes were calculated (Table 10).

After adjustment, it was found that the critical path of MOI had not changed. In contrast, there were noticeable changes for both MOA and MOC. Specifically, the complex and intermediate solutions for MOI were both “RH * ~GSC * GSD * INS”, and the solution consistency and solution coverage were 0.96 and 0.47, respectively. However, the parsimonious solution formulas were “~GSC * GSD” and “~GSC * INS”, and needed to be selected by the researchers. It is obvious that the explanatory power of the adjusted intermediate solution is relatively stronger, and we regard this solution as the explanatory path for MOI with low resource heterogeneity, weak governmental control, strong centralization and strong normative effectiveness. As for MOA, the adjusted intermediate solution and the parsimonious solution were “RH * GSC * INS”, which is different from “RH * GSC * GSD * INS” in Table 9. At the same time, the solution consistency decreased to 0.77, whereas the solution coverage remained as before. Clearly, the former solution’s explanatory power is stronger than the latter one. As shown in Table 9 and Table 10, the intermediate solution and complex solution of MOC changed from “~RH * ~GSC * ~GSD * ~INS” to “~GSC * ~GSD * ~INS” by adjusting the cross point of 0/1, the consistency value grew from 0.82 to 0.86, and the coverage value increased from 0.52 to 0.68. Obviously, the adjusted path of MOC demonstrates stronger explanatory power than the former one.

## 5. Discussion

### 5.1. Explanation of the Necessary Conditions

As shown in Table 6, strong governmental control is the necessary condition when selecting MOA, and centralization is the necessary and sufficient condition of MOA. It is worth noting that a centralized internal relationship, as well as the governmental control representing strong organizational and financial support, is needed to implement unified but limited management of health service providers when the original institutional setting and administrative institutions remain in the context of a bureaucratic health system. This is because the allocated health resources are affected by the path-dependence of the administrative hierarchies, which determine the leading role played by health authorities, and even the whole county-level government. In this sense, supply-driven health system reform in the form of the implementation of the Administration-oriented Organization Mode cannot be achieved without the transformation of government functions and government empowerment to IHC.

### 5.2. Explanation of the Sufficient Configurations

This research reveals three critical paths, i.e., three configurations in the process of IHC mode selection. Firstly, the explanatory path for MOI is low resource heterogeneity, weak governmental control, centralization and normalization, meaning that practitioners should place limits on externalized control and encourage member organizations to independently a relatively balanced internal interest mechanism in which the institutional environment has already been perfected, to some extent. Secondly, the explanatory path for MOA is low resource heterogeneity, strong governmental control, centralization, and normative effectiveness, and the truth table analyses indicated that health service providers should strive for governmental support, including organizational support and fiscal support, and adjust the internal power structure to achieve a unified management process when adopting MOA. Thirdly, the critical path for MOC is weak governmental control, weak centralization and weak normative effectiveness, and this confirms that most MOCs represent voluntary cooperations. In fact, the governmental control represented by the organizational and financial support in counties implementing MOC was also relatively weak, which can be partially attributed to the absense of health authorities or insufficient administrative coordination ability on the part of health authorities. Meanwhile, the absence of variable “~RH” in the second round truth table analysis indicates that diversified cooperation should be implemented in order to improve the medical conditions for health service providers at the township level in the present stage. However, we cannot declare that there is no need to consider the fundamental effect of resource heterogeneity when selecting MOC. Taken together, these three critical paths work against the proposed hypothesis claiming that a relatively more integrative organizational mode will be incompatible with high resource heterogeneity, strong governmental control, centralization and strong normalized institutional environment, and vice versa. Instead, these three paths indicate distinctive paths in IHC mode selection.

### 5.3. Differential Interpretation of Factors Affecting IHC Mode Selection

In this section, we interpret the explanatory factors that affect IHC mode selection. Firstly, our study suggests that MOI and MOA are accompanied by a low degree of resource heterogeneity among member organizations, while MOC is compatible with a high degree of resource heterogeneity or the absence of resource heterogeneity. This deviates from hypothesis 1, which was that the greater the resource heterogeneity among member organizations, the more likely member organizations would be to choose a relatively more integrative organizational mode, and vice versa. This phenomenon can be partially explained by the fact that both the voluntary and loose cooperation of members in MOC, and the policy implementation of MOA and MOI are practices of vertical integration among health service providers within a county. It can also be inferred that the fundamental effect of resource heterogeneity is a weakening in the specific implementation of IHC in the context of a bureaucratic health system.

Secondly, the study also illustrates the directive effects of governmental control and centralization. It is confirmed that a relatively more integrative organizational mode is formed in accordance with a greater degree of governmental control and a greater degree of centralization in the context of China’s authoritarian regime. Specifically, strong governmental control has a greater influence on the selection of MOA, and centralization has more influence over the choice of MOA and MOI. This is because MOC mainly refers to cooperation among medical businesses, and can be quickly achieved; MOA is mainly concerned with the distribution of human, property and material resources and requires strong governmental support, as well as empowered professional management institutions; and MOI mainly involves balancing internal interests, and the corresponding governance strategy is to explore a balanced mechanism that will gradually weaken the external control exerted by the health authority at the county-level, and even that of the whole government.

Furthermore, our study finds that the institutional environment demonstrates a significant influence over the selection of organizational mode, with different modes suggesting different requirements with respect to the normative effectiveness in the institutional environment. For instance, MOCs are social products of a weak regulatory institutional environment, while MOA and MOI are usually implemented in the context of a strong regulatory institutional framework. This finding proved that a more tightly integrated organizational mode is incompatible with more standardized institutional environments, and vice versa. Based on the arguments for institutionalism, the policies and related institutional arrangements that IHCs are reliant on can affect the mode selection in the context of bureaucratic health systems. To this end, if practitioners are determined to change the organizational mode at the county level, it will be necessary to change the existing property rights systems of hospitals, the medical personnel system, and the medical pricing system, etc., as well.

As described above, the insurance-driven organization mode (MOI) and the administration-oriented organization mode (MOA) are accompanied by lower degrees of resource heterogeneity among the member organizations, and it is unexpected that the contractual organization mode (MOC) would be compatible with higher degrees of resource heterogeneity, or the absence of this variable. In addition, strong governmental control has a greater influence on the selection of MOA than the other two modes, and centralization has greater influence over the choice of MOA and MOI. Moreover, we find that the institutional environment demonstrates an influence over the selection of organizational mode, with different modes suggesting different requirements with respect to normative effectiveness within the institutional environment.

## 6. Conclusions

In this study, the researchers aimed to explore the mechanism of mode selection in the implementation of IHC, as well as to confirm the causal conditions under which those selections may have been made, with regard to aspects such as resource heterogeneity, power structure and institutional normalization, and various configurations thereof, by conducting a Fuzzy-Sets Qualitative Comparative Analysis. Our study developed a theoretical framework consisting of resource heterogeneity, governance structure, normative effectiveness, and their various configurations. This framework is highly comprehensive, and is of great theoretical and practical significance. In fact, the Chinese health system has been undergoing profound institutional changes, and many complex problems have arisen in the practical implementation of IHC. IHCs have flourished in most counties, with the organizational modes that have been implemented in other regions rarely having been replicated in rural China. The organizational modes that have already been implemented differ in terms of their level of integration as a result of their individual conditions. Under such circumstances, this study comprehensively analyzes the mode selection mechanism of IHC mode selectionwith respect to three different aspects, and initially finds the critical paths for the three different organization modes. Our study also identifies that strong governmental control and centralization are necessary conditions in the selection of MOA, while finding that the three critical paths that lead to each organizational mode are sufficient to explain the actual mechanism in the bureaucratic environment of the healthcare system. In short, this study demonstrated the necessity of promoting implementation of IHC mode in consideration of the complex relationships among the causal conditions and their configurations under the bureaucratic conditions of the health system. In addition, this is of significance with respect to health system reform and the selection of IHC modes.

However, although the theoretical framework and the empirical verification of this study clearly explains how resource heterogeneity, governance structure and normative effectiveness influence the selection of IHC modes, our research has certain limitations. Admittedly, although acceptable, there are only two samples of MOI, due to the limited access to the health system in rural China. In addition, the internal selection mechanism and effects of different IHC organizational modes at different times or in different regions has been left for future research due to time limitations. More specifically, new variables, such as the age of the IHC, the degree of formalization, organizational behavior, and similar factors, still need to be explored in future study. Furthermore, mixed research methods should be employed. For instance, researchers can use the diachronic comparative research method to collect data at different stages when conducting case studies. This could help in exploring the relationships among IHC modes and resource heterogeneity, governance structure, and normative effectiveness at different stages of development. In addition, the effects of mode selection should be tested by analyzing the actual internal selection mechanisms of IHC modes, and it is necessary to examine the effects of different IHC modes under different configurations in future study to accurately guide IHC mode selection in practice. Furthermore, the study should be tested in a broader region, including other authoritarian and some non-authoritarian districts.

## Figures and Tables

**Table 1:** Classification and explanation of IHC modes.

Modes	Representation Form	Explanation
MOI	Medical insurance fund acts as the interest bond.	The funds are packaged and managed by the county-level hospital, and interest bonds are established among health service providers in a IHC.
MOA	The council (or other agencies) contacts health service providers.	Agencies are established to direct the standardization of medical business in an IHC and a unified but limited management is implemented.
MOC	Contracts or informal agreements are signed to complete cooperative tasks.	Contractual relationships are established among health service providers in an IHC.

Source: this table is compiled according to literature review and IHC implementation in rural China.

**Table 2:** Dimensions and interpretation of resource heterogeneity.

Dimensions	Description	Indicators
Service resources heterogeneity	Mainly include prevention, treatment and rehabilitation health services.	Number of sick beds, physicians (practitioners and assistant practitioners).
Technical resources heterogeneity	Mainly include equipment used for medical and pathology examination, etc.	Computerized tomography, magnetic resonance imaging, laboratory equipment, etc.

Source: this table is compiled by the authors according to relevant literature and practice.

**Table 3:** Distribution of sample cases.

Regions	County	Mode	Features
Developed Regions	XWC	MOA	Two service networks under the management of the integrated administration, finance and drug office.
	XLJ	MOA	Two service networks under the management of the integrated administration, finance and drug office.
	XNX	MOC	An IHC was established centered on the “platform of medical service technology”.
	XCS	MOC	Two service networks ensuring relationships between the rights and obligations based on contracts.
	XLY	MOC	Construction of two IHCs with “medical business” acting as the link.
Underdeveloped Regions	XSS	MOI	An IHC with the “Urban and Rural Medical Insurance Fund” acting as the link was constructed.
	XPJ	MOI	An IHC with the “Urban and Rural Medical Insurance Fund” acting as the link was constructed.
	XSL	MOA	An IHC was established under the leadership of the County People Hospital.
	XXW	MOA	Two service networks under the management of the integrated administration, finance and drug office.
	XTJ	MOC	Construction of an IHC centered by “technical guidance”.

Source: the authors generated the table based on official materials and field survey.

**Table 4:** Analytical dimensions and measurement of variables.

**Outcome Variables**	Mode Types	Insurance-driven Org. Mode (MOI)	If yes, assign 1; or, assign 0.
		Administration-oriented Org. Mode (MOA)	If yes, assign 1; or, assign 0.
		Contractual Org. Mode (MOC)	If yes, assign 1; or, assign 0.
**Conditional Variables**	Resource Heterogeneity (RH)	Heterogeneity of number of licensed physicians (RH1)	Measurement is based on the degree of heterogeneity of licensed physicians and the value is calibrated.
		Heterogeneity of number of open sick beds (RH2)	Measurement is based on the degree of heterogeneity of open sick beds and the value is calibrated.
		Heterogeneity of medical equipments (RH3)	Measurement is based on the degree of heterogeneity of medical equipments and the value is calibrated.
	Governance Structure (GS)	Governmental Control in the construction of IHC (GSC)	According to the degree of the governmental control, the value is set to “1/0.67/0.33/0”.
		Centralization: the distribution of decision-making power (GSD)	According to the degree of the centralization, the value is set to “1/0.67/0.33/0”.
	Normative Effectiveness (INS)	Normative effectiveness of policy documents to implement IHC (INS)	The value is calibrated based on the occurrence frequency of the specified words.

Note: this table was compiled by the authors.

**Table 5:** Measurement and value of governmental control and centralization.

Variables	Value	Measurement
Governmental control (GSC)	1	A working group is established with the head of county-level government acting as the leader, and special reform funds are set by the finance department.
	0.67	A working group is established under the leadership of the main government officials (often referred to as the permanent member of a committee in China).
	0.33	A working group is established under the leadership of the health department.
	0	Voluntary cooperative network is established among health service providers in county.
Centralization (GSD)	1	It has appointment power, funding allocation power and medicine & equipment procurement power simultaneously.
	0.67	It has any two of the rights mentioned above.
	0.33	It has any one of the rights mentioned above.
	0	It does not have appointment power, funding allocation power or medicine & equipment procurement power.

Note: this table was compiled by the authors.

**Table 6:** Analysis of the necessity of single causal conditions.

Causal Conditions	Consistency			Coverage		
	MOI	MOA	MOC	MOI	MOA	MOC
RH	0.50	0.67	0.37	0.13	0.58	0.28
GSC	0.50	1.00	0.28	0.10	0.73	0.17
GSD	0.67	1.00	0.11	0.14	0.78	0.07
INS	0.50	0.72	0.14	0.14	0.73	0.12

Note: the consistency formula for the results shown in Table 6 indicates the necessity, and the formula is: $(Y_{i}\leq X_{i})=\sum \left[\min(X_{i},Y_{i})\right]/\sum \left(Y_{i}\right)$.

**Table 7:** Analysis of combinations of causal conditions of MOC.

Elements Paths	MOC1	MOC2	MOC3	MOC4	MOC5	MOC6	MOC7	MOC8	MOC9	MOC10
RH	○		○	○			○		○	○
GSC	○	○	○		○		○	○	○	
GSD	○	○		○		○	○	○		○
INS	○	○	○	○	○	○				
Consistency	0.82	0.86	0.81	0.82	0.85	0.88	0.83	0.87	0.82	0.82
Coverage	0.52	0.69	0.52	0.52	0.69	0.79	0.53	0.73	0.53	0.53

Note: ○ indicates the negation of the causal conditions, and a space indicates the nonexistence of causal conditions.

**Table 8:** Analysis of combinations of causal conditions of MOA and MOI.

Elements Paths	MOA1	MOA2	MOA3	MOA4	MOA5	MOA6	MOA7	MOA8	MOA9	MOI1	MOI2	MOI3
RH	●		●	●			●		●	●	●	
GSC	●	●	●		●		●	●		○	○	○
GSD	●	●		●		●	●	●	●	●		
INS	●	●	●	●	●	●				●	●	●
Consistency	0.84	0.84	0.77	0.79	0.79	0.81	0.86	0.81	0.81	0.97	0.97	0.97
Coverage	0.54	0.72	0.54	0.54	0.72	0.72	0.65	1.00	0.65	0.81	0.81	0.81

Note: ● indicates the existence of the causal conditions, ○ indicates the negation of the causal conditions, and a space indicates the nonexistence of causal conditions.

**Table 9:** Different paths of IHC organizational modes (1).

	MOI	MOA	MOC
Intermediate Solution	RH*~GSC*GSD*INS	RH*GSC*GSD*INS	~RH*~GSC*~GSD*~INS
Solution Coverage	0.47	0.54	0.51
Solution Consistency	0.96	0.84	0.82

Note: “~” indicates the negation of the conditions.

**Table 10:** Different selection paths of IHC organization modes (2).

	MOI	MOA	MOC
Intermediate Solution	RH*~GSC*GSD*INS	RH*GSD*INS	~GSC*~GSD*~INS
Solution Coverage	0.47	0.54	0.68
Solution Consistency	0.96	0.77	0.86

Note: “~” indicates the negation of the condition.

## Appendix A

**Table A1:** Characteristics of Sample Cases.

Name	Implementation	Location	GDP (Million)		Data Availability
XWC	Yes	Eastern CN	24,714.86	Developed regions	Yes
XLJ	Yes	Eastern CN	46,491.43	Developed regions	Yes
XNX	Yes	Central CN	109,385.39	Developed regions	Yes
XCS	Yes	Central CN	143,273.54	Developed regions	Yes
XLY	Yes	Central CN	130,498.54	Developed regions	Yes
XSS	Yes	Central CN	8,488.53	Underdeveloped regions	Yes
XPJ	Yes	Central CN	25,784.79	Underdeveloped regions	Yes
XSL	Yes	Western CN	5674.93	Underdeveloped regions	Yes
XXW	Yes	Eastern CN	15,331.04	Underdeveloped regions	Yes
XTJ	Yes	Central CN	25,077.41	Underdeveloped regions	Yes

Source: the authors organized the table according to official materials.

## Appendix B

**Table A2:** Data Matrix.

CASE-ID	MO			RH	GSC	GSD	INS
	MOI	MOA	MOC				
XWCF	0	1	0	0.99	1	1	1
XWCS	0	1	0	0.07	1	1	1
XLJF	0	1	0	0.99	1	1	0.95
XLJS	0	1	0	0.62	1	1	0.95
XNXQ	0	0	1	0.39	0.33	0	0.11
XCSF	0	0	1	0.68	0.33	0	0.15
XCSS	0	0	1	0.95	0.33	0	0.15
XSSQ	1	0	0	0.39	0.67	0.67	0.39
XPJS	1	0	0	0.62	0.33	0.67	0.61
XSLQ	0	1	0	0.62	1	1	0.39
XXWF	0	1	0	0.45	1	1	0.39
XXWS	0	1	0	0.78	1	1	0.39
XTJF	0	0	1	0.15	0	0	0.22
XLYS	0	0	1	0.02	0.33	0.33	0.11
XLYS	0	0	1	0.01	0.33	0.33	0.11

Note: this sheet was compiled on the basis of Table 4 and Table 5.
